# Intraoperative Stress Burden, Adapted Textbook Outcome, and Overall Survival After Curative-Intent Gastrectomy for Gastric Adenocarcinoma: A Single-Center Retrospective Cohort Study

**DOI:** 10.3390/cancers18121975

**Published:** 2026-06-18

**Authors:** Jianfeng Li, Songyao Chen, Hui Ren, Jingyao Chen, Mingzhe Li, Wenhui Wu, Dongjie Yang, Changhua Zhang, Yulong He

**Affiliations:** 1 Digestive Medicine Center, The Seventh Affiliated Hospital of Sun Yat-sen University, Shenzhen 518107, China; lijianfeng@sysush.com (J.L.);; 2 Guangdong Provincial Key Laboratory of Digestive Cancer Research, Digestive Diseases Center, The Seventh Affiliated Hospital of Sun Yat-sen University, Shenzhen 518107, China

**Keywords:** stomach neoplasms, gastrectomy, intraoperative care, treatment outcome, survival analysis

## Abstract

Background/Objectives: textbook outcome (TO) is an established surgical quality measure, but failure is recognized only after postoperative events. We evaluated whether intraoperative stress burden (blood loss, fluid administration, and transfusion) is associated with adapted TO attainment and overall survival (OS) after curative-intent gastrectomy. Methods: in 2352 patients with gastric adenocarcinoma (2010–2020), an intraoperative stress burden score summed three binary components (blood loss > 200 mL, fluid > 68 mL/kg, and any transfusion) and was categorized as low, intermediate, or high. Adapted TO required R0 resection, ≥15 retrieved nodes, no Clavien–Dindo ≥ III complication, no unplanned reoperation, no 30-day mortality, and length of stay ≤ 12 days. Multivariable logistic and Cox models, overlap weighting, and sensitivity analyses were applied. Results: the median age was 60 years; 66.9% were male, 67.7% had pT3–4 and 42.0% pN2–3 disease, and 30.1% underwent minimally invasive surgery. TO attainment declined with increasing burden (78.0%, 70.5%, and 65.2%; *p* < 0.001). Intermediate and high burden were associated with TO failure (adjusted odds ratios 1.50 and 1.68), though the high-burden association was attenuated after adjusting for operative time. High burden was associated with worse OS (adjusted hazard ratio 1.36; 95% CI 1.15–1.62; 1.44 after overlap weighting). Risk was time-varying—strongest in the first postoperative year (HR 2.03), persisting at 12–60 months (HR 1.54), and absent beyond 60 months. Conclusions: higher intraoperative stress burden identified patients with lower adapted TO attainment and increased early mortality after gastrectomy. External validation is needed.

## Simple Summary

Intraoperative events during gastrectomy, such as blood loss, fluid administration, and transfusion, may reflect surgical complexity and physiological stress before postoperative complications become evident. In this retrospective cohort of 2352 patients undergoing curative-intent gastrectomy for gastric adenocarcinoma, we combined these three intraoperative factors into a simple intraoperative stress burden score. Higher burden was associated with lower adapted textbook outcome attainment and worse overall survival, especially within the early postoperative period. This score may help surgical and perioperative teams identify high-risk patients at the end of surgery and consider intensified postoperative monitoring, recovery support, and oncologic-care coordination. External validation is needed before routine clinical use.

## 1. Introduction

The quality evaluation of curative gastrectomy for gastric cancer is shifting from whether complete resection was achieved to whether long-term oncologic benefit is attained at acceptable perioperative cost. Although radical gastrectomy remains a cornerstone of curative treatment, postoperative recovery and long-term survival remain heterogeneous [1,2,3]. The textbook outcome (TO)—a patient-centered composite integrating R0 resection, adequate lymph node retrieval, freedom from major complications and unplanned reoperation, 30-day survival, and limited length of stay—has emerged as an established framework for evaluating surgical quality in gastric cancer [4,5]. Prior population-registry and cohort analyses have consistently shown that TO achievement is associated with better long-term survival [6,7,8,9], and the framework has since been extended to textbook oncological outcomes, chemotherapy adherence, and composite oncologic quality measures [10,11,12,13,14]. An upstream question thus emerges: which intraoperative processes might foreshadow postoperative quality and long-term survival risk before TO failure becomes apparent?

Two related evidence gaps are relevant. TO studies have largely treated TO as a postoperative quality “report card” or prognostic marker, whereas perioperative-exposure studies have typically analyzed intraoperative blood loss, fluid administration, and transfusion as isolated single factors. Single-exposure analyses are easier to interpret but do not capture the clinical reality of radical gastrectomy: increased blood loss frequently coincides with fluid resuscitation and transfusion decisions, and elevated fluid administration may reflect hemodynamic instability, prolonged operative time, or greater technical difficulty. Prior work has examined these exposures separately in relation to perioperative outcomes and long-term prognosis, but interpretation is often confounded by tumor burden, anemia, nutritional status, and operative complexity [15,16,17,18,19,20]. Although Enhanced Recovery After Surgery (ERAS) frameworks emphasize minimizing surgical stress and optimizing fluid therapy, most studies focus on short-term recovery, leaving the relationship between intraoperative resuscitation burden and long-term oncologic outcomes incompletely characterized [21,22,23].

To address this gap, we integrated three intraoperative exposures—high blood loss, high fluid administration, and any blood-product transfusion—into a simple, interpretable cumulative phenotype: the intraoperative stress burden (ISB). This phenotype is intended to capture the cumulative intraoperative insult arising from technical difficulty, hemodynamic perturbation, resuscitation requirements, and transfusion exposure. The term “stress burden” is used here as a clinical-operative descriptor of cumulative intraoperative perturbation and resuscitation burden, rather than as a direct physiological measurement of neuroendocrine or inflammatory stress. Compared with single-exposure indicators, the ISB score better aligns with surgeons’ clinical intuition of a “high-resuscitation case” or “technically challenging case.” Because the phenotype may simultaneously reflect tumor burden and operative complexity, we applied prespecified multivariable models and overlap weighting as a sensitivity analysis to assess robustness against measured baseline differences [24].

We therefore aimed to evaluate whether intraoperative stress burden is associated with adapted TO attainment and overall survival (OS) after curative-intent gastrectomy for gastric adenocarcinoma, and whether this association remains broadly consistent after sequential adjustment for downstream and concurrent variables. We hypothesized that ISB is associated with TO failure and, worse, OS; if confirmed, ISB could serve as an end-of-surgery risk-stratification signal, helping clinical teams identify patients requiring intensified postoperative monitoring, infection prevention, nutritional support, rehabilitation, and continuity of oncologic care.

## 2. Materials and Methods

### 2.1. Study Design and Subjects

**Figure 1:**
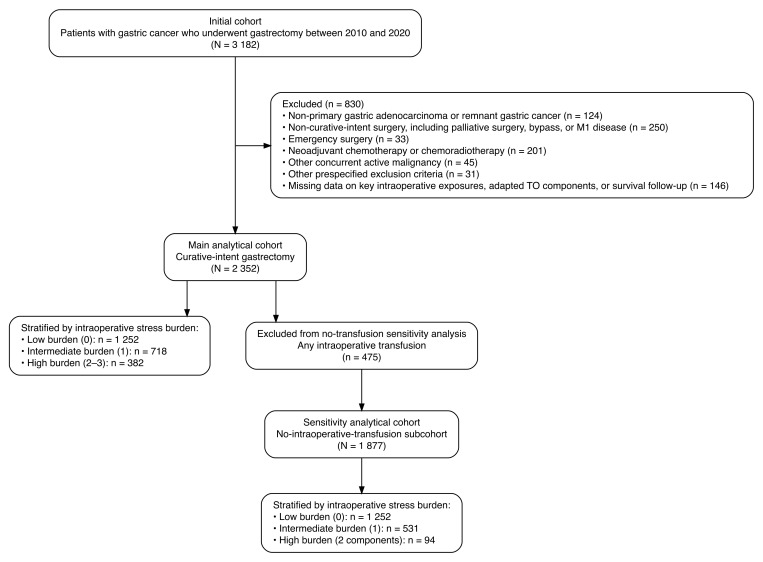
Flowchart of patient selection and analytical cohorts. TO, adapted textbook outcome.

This was a single-center retrospective cohort study conducted in accordance with the STROBE statement for observational studies [25]. Data were drawn from our institutional gastric cancer surgery database. All consecutive patients who underwent gastrectomy for gastric cancer between 2010 and 2020 were screened. Eligible patients had postoperatively confirmed primary gastric adenocarcinoma and underwent curative-intent gastrectomy, performed via open or minimally invasive (laparoscopic or robotic-assisted) approaches. Lymphadenectomy was performed according to the Japanese Gastric Cancer Association (JGCA) guidelines, with a standard extent of D2 or D2+. The patient selection flow is shown in Figure 1.

Inclusion criteria were pathologically confirmed as primary gastric adenocarcinoma; curative-intent gastrectomy; complete records of the three core intraoperative exposure variables (blood loss, fluid administration, and transfusion); complete perioperative outcome data sufficient to determine adapted TO; and documented survival status with date of last follow-up for OS analysis. Exclusion criteria were emergency surgery; palliative resection, bypass procedures, or exploratory laparotomy; distant metastases or intraoperative findings precluding curative resection; remnant gastric cancer; receipt of neoadjuvant therapy; another active malignancy; and missing data for any core variable.

A complete-case analysis was performed. Patients were not excluded on the basis of a minimum follow-up threshold; all patients with documented vital status and follow-up time were included in the OS analysis to avoid excluding early postoperative deaths. This study was approved by our institutional ethics committee, and informed consent was waived given the retrospective design and use of anonymized data. No imputation was used; complete data for the three core intraoperative exposures, all variables required to determine adapted TO, and vital status were required for inclusion, and patients with missing values in any core variable (*n* = 146) were excluded during cohort assembly (Figure 1).

### 2.2. Intraoperative Stress Burden Score

The intraoperative stress burden (ISB) score was constructed from three prespecified intraoperative exposures: high blood loss, high fluid administration, and transfusion. High blood loss and high fluid administration were each defined as exceeding the cohort-specific 75th percentile (blood loss > 200 mL; weight-adjusted fluid > 68 mL/kg)—an empirical stratification commonly used in observational cohorts and surgical benchmarking [26,27]; external applicability requires further validation. Intraoperative fluid administration was defined as the total crystalloid and colloid volume administered from anesthesia induction to skin closure, excluding blood products, and was normalized to actual body weight. Intraoperative transfusion was defined as receipt of any red blood cell or fresh frozen plasma transfusion administered from anesthesia induction to skin closure; platelet, cryoprecipitate, and albumin administration were not included. Transfusion was analyzed as a separate exposure component to avoid double-counting with fluid volume. Each exposure contributed 1 point, yielding a total score ranging from 0 to 3. Intraoperative transfusion followed a restrictive strategy rather than a separate written protocol, in keeping with standard surgical practice: red-cell transfusion was generally withheld above a hemoglobin of 100 g/L and considered below 70 g/L, while within the 70–100 g/L range, the decision was individualized by the attending anesthesiologist and surgeon according to cardiopulmonary reserve, age, hemodynamic status, the rate and volume of ongoing blood loss, and intraoperative arterial blood-gas findings, with a higher threshold (approximately 80 g/L) applied in elderly patients or those with cardiovascular comorbidity; fresh-frozen plasma was administered for coagulopathy or massive hemorrhage.

On the basis of total score, patients were classified into low (0 point), intermediate (1 point), and high (2–3 points) burden groups. Scores of 2 and 3 were combined into a high-burden category to reflect the cumulative risk state of multiple coexisting intraoperative exposures and to avoid unstable estimates due to small numbers in the top score category. Postoperative or non-intraoperative perioperative transfusions were not included in the score, preserving temporal consistency of the exposure definition and avoiding contamination of an intraoperative risk phenotype with postoperative events. Detailed definitions of adapted TO and the ISB score are provided in Supplementary Table S1.

### 2.3. Outcomes

The primary outcomes were attainment of adapted textbook outcome (TO) and OS. The adapted TO definition drew on prior TO frameworks in upper gastrointestinal and gastric cancer studies and was refined to reflect data availability and the local postoperative recovery pathway [4,5,6,13,14]. Patients were considered to have achieved adapted TO only when all six criteria were met: microscopically margin-negative (R0) resection, retrieval of at least 15 lymph nodes, absence of Clavien–Dindo grade III or higher postoperative complications, no unplanned reoperation, survival beyond 30 days after surgery, and postoperative length of stay of 12 days or less. R0/R1/R2 status was determined by the institutional pathology department per the American Joint Committee on Cancer (AJCC) 8th edition, with assessment of proximal, distal, and (where applicable) circumferential margins; patients with intraoperatively determined unresectable disease had already been excluded by the exclusion criteria, and the resulting high R0 rate is consistent with stringent preoperative selection for resectability.

Length of stay ≤ 12 days was used to define absence of prolonged postoperative stay; this threshold corresponded to the 75th percentile of postoperative length of stay in our cohort and was chosen to reflect the local recovery pathway rather than directly adopting the 21-day threshold commonly used in Western TO studies. Because the institutional database did not systematically record 30-day readmission, this variable was not incorporated into TO. Severe postoperative complications were defined as Clavien–Dindo grade III or higher, capturing major morbidity requiring surgical, endoscopic, radiologic, or critical-care intervention [28]. Patients meeting all six criteria were classified as having achieved TO; failure of any single criterion constituted TO failure.

Overall survival was defined as the interval from the date of surgery to all-cause death; patients still alive were censored at their last follow-up. In extended Cox models, TO was included as a conditional covariate to assess whether the high-burden–OS association remained directionally stable; this is not interpreted as a formal mediation analysis.

### 2.4. Covariates

Covariates in the multivariable models were prespecified on the basis of clinical relevance, prior literature, and each variable’s position on the causal pathway, rather than being screened by univariate *p* values. The primary model included sex, age, body mass index (BMI), American Society of Anesthesiologists (ASA) physical status, preoperative serum albumin, preoperative hemoglobin, Borrmann type, tumor location, differentiation grade, pathologic T stage, pathologic N stage, surgical approach, and type of gastrectomy. Pathologic T and N stages were classified according to the AJCC 8th edition gastric cancer staging system [29]. The extended Cox Model 3 additionally included receipt of adjuvant chemotherapy and lymph node ratio (LNR; metastatic nodes/total retrieved, per 0.1 increase) as descriptive extended covariates.

ISB component variables were not entered as separate covariates in the primary models to avoid duplicate adjustment. Postoperative complications, length of stay, individual TO components, and other variables temporally posterior to the intraoperative exposure were likewise excluded from the primary models to reduce over-adjustment.

### 2.5. Statistical Analysis

Continuous variables were summarized as medians with interquartile ranges or as means with standard deviations, as appropriate. Categorical variables were summarized as frequencies and percentages. Differences across the three intraoperative stress burden groups were assessed using Pearson’s chi-square test or Fisher’s exact test for categorical variables and the Kruskal–Wallis test for continuous variables. All statistical tests were two-sided, and *p* < 0.05 was considered statistically significant.

The primary analyses used multivariable logistic regression to evaluate the association between intraoperative stress burden and TO failure, and Kaplan–Meier estimation and multivariable Cox proportional-hazards models to evaluate its association with OS, with the low-burden group as the reference. Tests for trend were calculated by entering ISB as an ordinal variable (coded 0/1/2). The OS analysis built three sequential Cox models: Model 1 adjusted for the prespecified baseline covariates (primary analysis); Model 2 additionally included TO status; and Model 3 additionally included receipt of adjuvant chemotherapy (yes/no) and LNR (per 0.1 increase). Models 2 and 3 are descriptive extended models intended to assess whether the high-burden–OS association remained directionally stable after sequential inclusion of downstream or concurrent variables; they are not interpreted as formal mediation analyses or causal effect estimates.

Prespecified sensitivity and exploratory analyses included the following: (1) overlap weighting was applied to estimate the average treatment effect in the overlap population (ATO) [24], with generalized propensity scores estimated by multinomial logistic regression and 95% confidence intervals derived from robust standard errors after weighting; (2) the primary models were further adjusted for operative time (per 30 min) and surgical era (2010–2013, 2014–2017, and 2018–2020) to assess sensitivity to operative complexity and era effects; (3) Cox analyses were repeated in patients who did not receive intraoperative transfusion; (4) the original 0–3 ISB score was reported separately to evaluate dose–response; (5) the proportional-hazards assumption was tested using Schoenfeld residuals, and when violated, Cox analyses were repeated within prespecified time intervals (0–12, 12–60, and >60 months); (6) five-year time-dependent C-statistics were compared across three nested models—baseline covariates, baseline plus the ISB score, and baseline plus the three components entered separately—to assess incremental discrimination; and (7) subgroup analyses were performed by pT stage, pN stage, surgical approach, and receipt of adjuvant chemotherapy, with interaction *p* values reported. Detailed results are presented in Supplementary Tables S2–S11 and Supplementary Figure S6. Sensitivity analyses, time-interval analyses, subgroup analyses, and discrimination analyses were considered exploratory; no adjustment for multiple comparisons was performed, and *p* values from these analyses should be interpreted descriptively. All statistical analyses were performed using R version 4.5.2 (R Foundation for Statistical Computing, Vienna, Austria), with the survival, WeightIt, cobalt, survC1, timeROC, and sandwich packages, among others. As additional sensitivity analyses, we also (8) compared the incremental discrimination of the ISB score with operative time using nested Cox and logistic models and likelihood-ratio tests, and (9) reconstructed the ISB score using alternative thresholds (median, 90th percentile, and fixed clinically oriented cutoffs) and repeated the primary OS analysis (Supplementary Tables S12 and S13). We additionally compared the composite ISB score with its three individual components (high blood loss, high fluid administration, and any intraoperative transfusion) entered simultaneously, using nested models, likelihood-ratio tests, and mutually adjusted component associations (Supplementary Table S14).

## 3. Results

### 3.1. Study Cohort and Baseline Characteristics

During the 2010–2020 study period, 3182 patients who underwent gastrectomy for gastric cancer at our center were screened for eligibility. After applying the prespecified exclusion criteria, 830 patients were excluded, leaving 2352 patients with gastric adenocarcinoma who underwent curative-intent gastrectomy in the main analytical cohort; of these, 1252 were classified as low burden, 718 as intermediate burden, and 382 as high burden. The no-intraoperative-transfusion sensitivity cohort included 1877 patients (low burden, 1252; intermediate burden, 531; high burden, 94). The patient-selection flow is shown in Figure 1.

Distinct baseline and operative differences were observed across ISB groups. Compared with the low burden group, patients in the high burden group were older, had lower preoperative serum albumin and hemoglobin, and higher proportions of elevated carcinoembryonic antigen (CEA) and cancer antigen 125 (CA125), pT3–4 and pN2–3 disease, open surgery, greater intraoperative blood loss, longer operative time, and greater fluid administration. Complete baseline characteristics are presented in Table 1. These differences indicate that elevated ISB reflects a high-risk scenario in which baseline patient status, tumor burden, and operative complexity converge.

### 3.2. Intraoperative Stress Burden and Perioperative Outcomes

**Figure 2:**
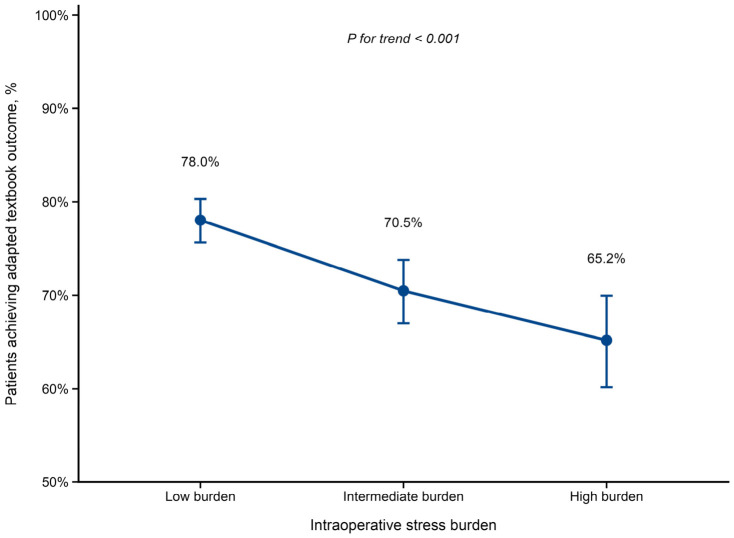
Association between intraoperative stress burden and adapted textbook outcome attainment. Points represent adapted textbook outcome attainment rates; error bars indicate 95% confidence intervals. *p* for trend was calculated across ordered burden groups.

The overall adapted TO attainment rate was 73.6% (1732/2352). TO attainment declined stepwise with rising intraoperative stress burden: 78.0% (977/1252) in the low burden group, 70.5% (506/718) in the intermediate burden group, and 65.2% (249/382) in the high burden group (*p* < 0.001; Figure 2). The absolute reduction of 12.8 percentage points from the low- to the high-burden group indicates a clear clinical gradient between intraoperative stress burden and overall postoperative quality.

Adverse perioperative outcomes followed the same direction. Overall complication rates increased from 15.3% in the low-burden group to 18.1% in the intermediate-burden group and 24.1% in the high-burden group (*p* < 0.001). Rates of severe complications were 6.9%, 8.8%, and 11.0%, respectively (*p* = 0.032), and rates of infectious complications were 9.6%, 12.8%, and 18.8%, respectively (*p* < 0.001). The median postoperative length of stay was 8, 9, and 10 days, respectively (*p* < 0.001). The proportion of R1/R2 resections was higher in the high-burden group, whereas the total number of retrieved lymph nodes did not differ significantly across groups (cohort-wide median, 37 nodes; IQR, 27–49), a yield consistent with that expected under D2 or D2+ lymphadenectomy. Detailed perioperative outcomes are provided in Table 2.

### 3.3. Intraoperative Stress Burden and TO Failure

After adjustment for the prespecified baseline covariates, the intraoperative stress burden remained significantly associated with TO failure. Using the low-burden group as the reference, both the intermediate- and high-burden groups had significantly elevated TO failure risk (adjusted odds ratio [aOR], 1.50; 95% CI, 1.19–1.87 and 1.68; 95% CI, 1.28–2.21, respectively; *p* for trend < 0.001; Table 3). In a prespecified sensitivity analysis additionally including operative time, the association in the high-burden group was attenuated and lost statistical significance (aOR 1.22; 95% CI, 0.90–1.64), and the association in the intermediate-burden group was similarly attenuated (aOR 1.26; 95% CI, 1.00–1.60); operative time itself was independently associated with TO failure (per 30-min increase, aOR 1.12). Sensitivity analyses additionally adjusting for surgical era were directionally consistent (Supplementary Tables S5 and S6).

These findings indicate that, although ISB was significantly associated with TO failure in the primary model, the association overlapped substantially with operative time; the score should be interpreted as a parsimonious composite marker of intraoperative complexity and resuscitation burden, rather than as an operative-time-independent predictor of TO failure.

### 3.4. Intraoperative Stress Burden and Overall Survival

At the end of follow-up, the cohort-wide median follow-up was 88.0 months (95% CI, 85.7–90.0), estimated by the reverse Kaplan–Meier method; a total of 1061 deaths occurred. The 5-year overall survival rates were 72.0% (95% CI, 69.5–74.7), 64.2% (95% CI, 60.6–67.9), and 49.6% (95% CI, 44.5–55.2) in the low-, intermediate-, and high-burden groups, respectively.

**Figure 3:**
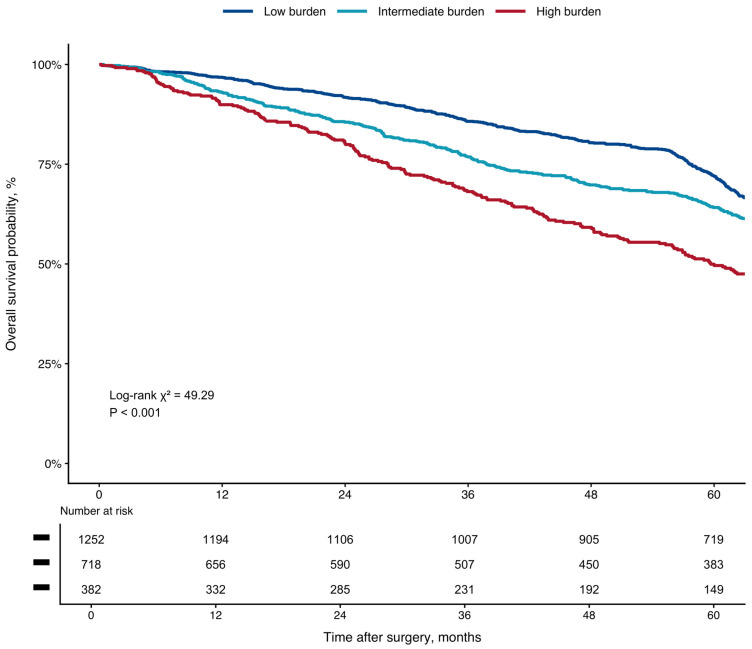
Overall survival according to intraoperative stress burden after curative-intent gastrectomy. Curves are stratified by low, intermediate, and high intraoperative stress burden. The risk table shows the number of patients at risk. Survival distributions were compared using the log-rank test.

Kaplan–Meier curves showed clear separation in overall survival across the intraoperative stress burden groups, with the high-burden group having the lowest survival, the intermediate-burden group intermediate, and the low-burden group the highest (log-rank χ^2^ = 49.29, *p* < 0.001; Figure 3).

In multivariable Cox models, the OS risk difference was concentrated in the high burden group. After adjustment for the prespecified covariates in Model 1, the intermediate burden group did not differ significantly from the low-burden group (adjusted hazard ratio [aHR], 1.07; 95% CI, 0.93–1.24; *p* = 0.33), whereas the high-burden group had a significantly elevated mortality risk (aHR, 1.36; 95% CI, 1.15–1.62; *p* < 0.001; *p* for trend < 0.001).

In Model 2, which additionally included TO status, the high-burden association with worse OS persisted (aHR, 1.34; 95% CI, 1.13–1.59; *p* < 0.001; *p* for trend = 0.002), and the intermediate burden group again did not reach statistical significance (aHR, 1.05; 95% CI, 0.91–1.22; *p* = 0.49). Failed TO was itself associated with worse OS (aHR, 1.27; 95% CI, 1.11–1.46; *p* < 0.001). Kaplan–Meier curves stratified by TO status are shown in Supplementary Figure S1.

In the extended Model 3, which additionally included receipt of adjuvant chemotherapy and LNR, the high-burden association with worse OS persisted (aHR, 1.32; 95% CI, 1.12–1.57; *p* for trend = 0.003). Receipt of adjuvant chemotherapy was associated with lower mortality risk (aHR, 0.82; 95% CI, 0.72–0.94), and higher LNR (per 0.1 increase) was associated with higher mortality risk (aHR, 1.09; 95% CI, 1.07–1.12). The high-burden association with OS remained directionally stable across Models 1, 2, and 3 (aHR 1.36 → 1.34 → 1.32), and was unchanged in sensitivity analyses additionally adjusting for operative time or surgical era (Supplementary Tables S5 and S6). Complete Cox model results are presented in Table 4; a forest plot is provided in Supplementary Figure S2.

The primary findings were directionally consistent across prespecified sensitivity analyses. ATO overlap-weighted Cox analysis confirmed a stable high-burden association with worse OS (HR 1.44; 95% CI, 1.21–1.73; Supplementary Tables S2–S4 and Supplementary Figures S4 and S5). The association remained directionally unchanged after additional adjustment for operative time and surgical era (Supplementary Tables S5 and S6), exhibited a dose–response gradient when the original 0–3 ISB score was modeled separately (Supplementary Table S7), and was directionally consistent in the no-intraoperative-transfusion subgroup analysis (Supplementary Figure S3). Schoenfeld residual testing indicated a violation of the proportional-hazards assumption for the primary exposure (Supplementary Table S8); time-interval-stratified Cox analyses showed that the high-burden association with OS was concentrated in the first postoperative year (0–12 months: HR 2.03), was attenuated but persisted at 12–60 months, and was no longer present beyond 60 months (Supplementary Table S9). In 5-year time-dependent C-statistic evaluation, the addition of the categorical ISB score to the baseline covariates produced only a small improvement in discrimination (0.739 → 0.743), slightly below the model in which the three components were entered separately (0.744; Supplementary Table S10). Subgroup analyses showed that the association was more pronounced in patients with pT3–4 disease, those with pN2–3 disease, and those not receiving adjuvant chemotherapy, with all interaction *p* values > 0.05 (Supplementary Table S11 and Supplementary Figure S6). In head-to-head analyses (Supplementary Table S12), ISB provided additional prognostic information for OS beyond operative time (likelihood-ratio χ^2^ = 9.90, *p* = 0.007; high-burden HR 1.34, 95% CI 1.12–1.60), whereas operative time did not improve the OS model beyond ISB (*p* = 0.558) and was not independently associated with OS; for adapted TO, the converse was observed, with operative time dominating (*p* < 0.001) and ISB adding little beyond it (*p* = 0.126). The high-burden–OS association was directionally stable across alternative ISB thresholds (Supplementary Table S13). A full comparison of the composite score with its individual components, including mutually adjusted component-level associations, is provided in Supplementary Table S14; the composite did not discriminate better than its components, consistent with its intended role as a parsimonious summary rather than a maximally discriminating model.

## 4. Discussion

In this study, we propose intraoperative stress burden as an upstream, end-of-surgery clinical phenotype that complements adapted textbook outcome rather than replacing it. In this single-center cohort of 2352 patients undergoing curative-intent gastrectomy for gastric adenocarcinoma, three findings emerged. First, a higher intraoperative stress burden score was associated with lower adapted TO attainment. Second, this TO failure association attenuated substantially once operative time was added, suggesting that much of the ISB–TO signal reflects underlying operative complexity rather than an independent effect. Third, the association of ISB with worse overall survival was not eliminated by adjustment for TO status, adjuvant chemotherapy, lymph node ratio (LNR), operative time, or surgical era, and was strongest within the first postoperative year, attenuating but remaining evident through 12–60 months, and being no longer present beyond 60 months on time-stratified analysis. We interpret these results descriptively—as evidence that the cumulative intraoperative insult tracks with subsequent recovery and early mortality—rather than as proof of mediation or an independent causal effect.

These endpoint-specific findings further clarify the relationship between ISB and operative complexity. For adapted TO, the ISB signal overlaps substantially with operative time and is best interpreted as a composite marker of intraoperative complexity. For overall survival, by contrast, the burden signal was not explained by operative time, which added no further discriminatory information once ISB was included (Supplementary Table S12); this argues against ISB being merely a surrogate for a longer or technically more difficult operation with respect to long-term outcomes. Consistently with its small incremental discrimination, we position ISB not as a prediction model but as a simple, reproducible end-of-surgery marker for triage and intensified postoperative care, whose absolute thresholds were derived in this cohort and remained prognostic across alternative cut-points (Supplementary Table S13) but still require external recalibration and validation.

Prior gastric cancer TO studies have largely treated TO as a postoperative quality measure or prognostic marker: the PRESTO cohort, population-based analyses, and systematic reviews have consistently shown that TO achievement is associated with better long-term survival [6,7,8,9,13,14], and recent work has expanded this idea to textbook oncological outcomes, chemotherapy adherence, and composite oncologic-care quality [10,11,12,13,14]. In a separate line of evidence, an exploratory analysis of JCOG1001 and systematic reviews have linked postoperative complications to worse long-term survival [30,31,32], and studies of perioperative transfusion, intraoperative blood loss, and fluid management have similarly suggested that intraoperative blood loss and blood-product exposure are associated with long-term outcomes, although these associations are often confounded by baseline patient status, tumor burden, and operative complexity [15,16,17,18,19,20]. The contribution of the present study is to integrate these previously isolated intraoperative exposures into an upstream, end-of-surgery composite risk phenotype that complements—rather than replaces—the TO framework for postoperative quality evaluation, a positioning consistent with recent discussions of TO/TOO heterogeneity and composite surgical quality metrics [13,14]. Most recently, Marrelli et al. proposed a gastric-cancer-specific textbook outcome index (TOGS) refined from an Italian multicenter cohort [33], representing further evolution of the TO itself; our work is complementary, examining an upstream, end-of-surgery intraoperative signal that may identify patients at risk for TO failure rather than redefining the TO.

Increased blood loss, elevated fluid administration, and transfusion exposure are rarely isolated events; they tend to co-occur in cases of greater technical difficulty, hemodynamic perturbation, or heightened resuscitation requirements. Although ERAS guidelines for gastrectomy emphasize minimizing surgical stress and optimizing resuscitation, the associated strategies have typically been evaluated against short-term recovery endpoints [21,22,23]. Rather than attempting to isolate which exposure has an independent causal effect, we integrated them into a cumulative burden phenotype. Time-interval-stratified analyses showed that the high-burden–OS association was concentrated in the first postoperative year and was no longer present beyond 60 months; this temporal distribution supports the clinical hypothesis that the intraoperative insult acts through early postoperative complications, infection, and disrupted oncologic-care continuity—affecting early mortality—rather than through late tumor-specific mortality. In discrimination analyses, the addition of the ISB score yielded only marginal incremental discrimination over its three components; we therefore view the score as a simple composite clinical marker rather than a discrimination-maximizing prediction model. Subgroup analyses showed that the association was more pronounced in patients with pT3–4 disease, those with pN2–3 disease, and those not receiving adjuvant chemotherapy (all interaction *p* values > 0.05).

Clinically, ISB is a practical end-of-surgery marker that surgical, anesthetic, and perioperative care teams already have in hand by the time the patient leaves the operating room. In high-burden patients, clinical teams could intensify postoperative monitoring, infection prevention, nutritional support, rehabilitation, and adjuvant-therapy coordination earlier, while incorporating hemostasis control, goal-directed resuscitation, and judicious transfusion practices into broader quality-improvement efforts. ISB does not replace surgical judgment; rather, it standardizes the subjective impression of a difficult, high-resuscitation case into a reproducible, end-of-surgery handoff signal. Once externally validated, ISB may complement established perioperative risk-stratification tools by providing a simple end-of-surgery signal for enhanced-recovery pathway triage and adjuvant-therapy continuity.

These results sit within the growing textbook-outcome literature. Across gastric cancer cohorts, adapted TO attainment varies widely with the definition used and is consistently associated with better long-term survival, with advanced stage, greater comorbidity, more extensive resection, and postoperative complications being the principal determinants of failure [6,7,8,9,10,11,12,13,14]. The surgical approach also contributed in our cohort: minimally invasive resection was associated with higher adapted TO attainment than open surgery (81.1% versus 70.4%), consistent with randomized evidence that laparoscopic distal gastrectomy achieves oncologic outcomes comparable to open surgery while improving short-term recovery [34]; because the approach was not randomized, however, this comparison is susceptible to selection and should be interpreted cautiously (Section Limitations).

Several adjacent lines of work complement these findings. Enhanced-recovery principles are increasingly extended to the most demanding procedures, including cytoreductive surgery with hyperthermic intraperitoneal chemotherapy, where they shorten length of stay and may facilitate timely adjuvant therapy [35]. Dynamic perioperative changes in systemic inflammatory markers, such as the neutrophil-to-lymphocyte ratio, also carry prognostic information after gastrectomy and may partly reflect the same physiological stress that the ISB attempts to summarize [36]. Finally, health-related quality of life, assessed with instruments such as the FACT-Ga and SF-36v2 and influenced by the type of resection, tumor location, sex, and time since surgery, is now regarded as a core outcome of curative gastrectomy [37]; future ISB-informed care pathways should therefore track patient-reported recovery alongside survival and textbook outcome.

### Limitations

This study has several limitations. First, the design is single-center and retrospective, with a relatively long study period; although multivariable adjustment, ATO weighting, and surgical-era sensitivity analyses were applied, residual confounding related to operative complexity, anesthetic management, surgeon experience, era effects, and tumor biology cannot be excluded. Second, the ISB score was defined using cohort-specific 75th percentile thresholds (blood loss > 200 mL; fluid administration > 68 mL/kg), and the applicability of these thresholds in other centers and surgical settings requires external validation; the score’s incremental discrimination over its three components was limited, and it should be understood as a simple composite clinical marker rather than as a predictive model. Third, in this study, we used an adapted TO definition tailored to local data availability and recovery pathway, excluding 30-day readmission and unplanned intensive care unit admission, with the length-of-stay threshold set at the cohort-specific 75th percentile (12 days); the resulting TO attainment rate should therefore not be directly compared with studies using different TO definitions [13,14]. Because several components of the recently proposed gastric-cancer-specific TO index (TOGS) [33] were not systematically captured in our institutional database, we could not reconstruct the TOGS definition; future external validation should evaluate whether ISB can identify patients at risk of failure to achieve standardized TOGS and adverse long-term oncologic outcomes. Fourth, the granularity of outcome and treatment data was limited: only OS was available, with no information on disease-free or recurrence-free survival, cause of death, or recurrence patterns; adjuvant chemotherapy was recorded only as receipt versus non-receipt, without details on completed cycles, dose intensity, or time to initiation, and the receipt-versus-non-receipt classification may additionally be subject to immortal time bias and treatment-selection effects; and the number of high-burden patients in the no-intraoperative-transfusion subgroup was small (*n* = 94), with limited statistical power. Fifth, the proportional-hazards assumption was violated for the primary exposure and was addressed through time-interval-stratified analyses; the hazard ratios from Models 1–3 should be interpreted as average associations across the full follow-up period, distinct from the stronger associations observed within the first postoperative year, and the time-interval analysis is exploratory. Coefficients for surgical approach (open vs. minimally invasive) should not be interpreted causally because approach selection was non-random and may reflect tumor location, operative era, surgeon experience, and unmeasured patient-selection factors. Macroscopic tumor classification may be less standardized in early-stage (pT1) lesions; Borrmann type was therefore used as an adjustment covariate according to the available institutional database record. Moreover, because ISB is by design a marker at which baseline patient risk, tumor burden, and operative complexity converge, residual and unmeasured confounding (e.g., surgeon experience, anesthetic management, intraoperative hemodynamics, frailty, and tumor biology) cannot be excluded, and all reported associations should be interpreted as descriptive rather than causal. The high R0 resection rate reflects the way patients entered the cohort rather than an anomaly of outcome ascertainment: all cases were reviewed preoperatively by a multidisciplinary tumor board, and patients judged unlikely to achieve a margin-negative resection with upfront surgery—for example, those with bulky or locally advanced tumors—were preferentially triaged to neoadjuvant chemotherapy and were therefore excluded (receipt of neoadjuvant therapy was an exclusion criterion), as were patients found to have unresectable or metastatic disease at operation; the cohort thus represents a population stringently selected for upfront curative-intent resection, which constrains generalizability to unselected or locally advanced populations. In addition, overall survival was defined by all-cause death, and cause-specific mortality was not available; although unrelated deaths could in principle dilute the association, the concentration of the high-burden effect within the first postoperative year (Supplementary Table S9) argues against unrelated late mortality as the principal driver. Comorbidities were ascertained from documented medical history fields using prespecified keyword rules rather than a structured comorbidity instrument; therefore, conditions not explicitly recorded may have been under-ascertained. However, recorded comorbidity prevalence did not differ across ISB groups, and the high-burden association with survival was unchanged after additional adjustment for hypertension and diabetes.

## 5. Conclusions

In this single-center cohort, a higher intraoperative stress burden identified patients with lower adapted TO attainment and an elevated risk of death within the first postoperative year following curative-intent gastrectomy. Much of the TO failure association reflected operative complexity, whereas the early survival signal persisted after adjustment for TO status, adjuvant chemotherapy, LNR, operative time, and surgical era. These results require external confirmation in multicenter cohorts, and they raise a practical question for the field: whether perioperative strategies that reduce intraoperative stress burden translate into improved long-term outcomes.

## Figures and Tables

**Table 1:** Baseline clinicopathologic and perioperative characteristics according to intraoperative stress burden.

Characteristic	Overall (*N* = 2352)	Low Burden (*N* = 1252)	Intermediate Burden (*N* = 718)	High Burden (*N* = 382)	*p*-Value
Sex					0.042
Male	1573 (66.9%)	866 (69.2%)	461 (64.2%)	246 (64.4%)	
Female	779 (33.1%)	386 (30.8%)	257 (35.8%)	136 (35.6%)	
Age, years	60 (52, 67)	60 (51, 66)	60 (50, 67)	63 (55, 70)	<0.001
Body mass index, kg/m^2^	21.4 (19.1, 24.0)	22.0 (20.0, 24.0)	20.9 (19.0, 23.1)	21.0 (19.0, 23.0)	<0.001
ASA physical status					0.517
I–II	1877 (79.8%)	1009 (80.6%)	570 (79.4%)	298 (78.0%)	
III–IV	475 (20.2%)	243 (19.4%)	148 (20.6%)	84 (22.0%)	
Medical history					
Hypertension	440 (18.7%)	248 (19.8%)	118 (16.4%)	74 (19.4%)	0.170
Diabetes mellitus	182 (7.7%)	100 (8.0%)	47 (6.5%)	35 (9.2%)	0.269
Coronary heart disease	53 (2.3%)	27 (2.2%)	15 (2.1%)	11 (2.9%)	0.663
Heart failure	15 (0.6%)	9 (0.7%)	3 (0.4%)	3 (0.8%)	0.667
Chronic kidney disease	29 (1.2%)	16 (1.3%)	9 (1.3%)	4 (1.0%)	0.936
COPD or chronic bronchitis	23 (1.0%)	11 (0.9%)	10 (1.4%)	2 (0.5%)	0.330
Any comorbidity	589 (25.0%)	324 (25.9%)	161 (22.4%)	104 (27.2%)	0.131
Preoperative albumin, g/L	38.4 (35.5, 41.2)	39.0 (36.4, 41.8)	38.2 (35.0, 41.1)	36.5 (33.2, 40.1)	<0.001
Preoperative hemoglobin, g/L	125 (104, 139)	130 (115, 142)	121 (96, 135)	110 (86, 128)	<0.001
Preoperative CEA					0.006
Normal	1988 (84.5%)	1077 (86.0%)	608 (84.7%)	303 (79.3%)	
Elevated	364 (15.5%)	175 (14.0%)	110 (15.3%)	79 (20.7%)	
Preoperative CA125					<0.001
Normal	2254 (95.8%)	1217 (97.2%)	682 (95.0%)	355 (92.9%)	
Elevated	98 (4.2%)	35 (2.8%)	36 (5.0%)	27 (7.1%)	
Borrmann type					<0.001
Type I	101 (4.3%)	53 (4.2%)	32 (4.5%)	16 (4.2%)	
Type II	614 (26.1%)	392 (31.3%)	156 (21.7%)	66 (17.3%)	
Type III	1421 (60.4%)	730 (58.3%)	442 (61.6%)	249 (65.2%)	
Type IV	216 (9.2%)	77 (6.2%)	88 (12.3%)	51 (13.4%)	
Tumor location					<0.001
Upper	579 (24.6%)	298 (23.8%)	151 (21.0%)	130 (34.0%)	
Middle	477 (20.3%)	253 (20.2%)	156 (21.7%)	68 (17.8%)	
Lower	1142 (48.6%)	599 (47.8%)	377 (52.5%)	166 (43.5%)	
Whole stomach	154 (6.5%)	102 (8.1%)	34 (4.7%)	18 (4.7%)	
Differentiation grade					<0.001
Moderately/Well differentiated	1097 (46.6%)	614 (49.0%)	293 (40.8%)	190 (49.7%)	
Poorly/Undifferentiated	1255 (53.4%)	638 (51.0%)	425 (59.2%)	192 (50.3%)	
Pathologic T stage					<0.001
pT1–2	760 (32.3%)	505 (40.3%)	194 (27.0%)	61 (16.0%)	
pT3–4	1592 (67.7%)	747 (59.7%)	524 (73.0%)	321 (84.0%)	
Pathologic N stage					<0.001
pN0–1	1365 (58.0%)	773 (61.7%)	408 (56.8%)	184 (48.2%)	
pN2–3	987 (42.0%)	479 (38.3%)	310 (43.2%)	198 (51.8%)	
Lymph node ratio (LNR)	0.04 (0.00, 0.18)	0.03 (0.00, 0.16)	0.05 (0.00, 0.19)	0.08 (0.00, 0.27)	<0.001
Surgical approach					<0.001
Open	1644 (69.9%)	794 (63.4%)	560 (78.0%)	290 (75.9%)	
Minimally invasive (laparoscopic or robotic)	708 (30.1%)	458 (36.6%)	158 (22.0%)	92 (24.1%)	
Type of gastrectomy					0.001
Distal	1274 (54.2%)	711 (56.8%)	346 (48.2%)	217 (56.8%)	
Proximal	60 (2.6%)	37 (3.0%)	16 (2.2%)	7 (1.8%)	
Total	1018 (43.3%)	504 (40.3%)	356 (49.6%)	158 (41.4%)	
Adjuvant chemotherapy					0.052
No	1179 (50.1%)	655 (52.3%)	335 (46.7%)	189 (49.5%)	
Yes	1173 (49.9%)	597 (47.7%)	383 (53.3%)	193 (50.5%)	
Intraoperative blood loss, mL	100 (100, 200)	100 (50, 150)	150 (100, 250)	400 (300, 600)	<0.001
Operative time, min	285 (240, 340)	265 (225, 310)	300 (250, 350)	333 (280, 405)	<0.001
Intraoperative fluid administration, mL/kg	56 (45, 68)	50 (42, 58)	67 (52, 77)	72 (58, 85)	<0.001

Note: Values are median (Q1, Q3) or *n* (%). *p* values: Kruskal–Wallis for continuous variables; Pearson χ^2^ or Fisher’s exact (when expected count < 5) for categorical ones. LNR = metastatic ÷ total nodes retrieved. Minimally invasive includes laparoscopic and robotic cases. Lymph node dissection performed per JGCA guidelines (standard D2 or D2+). R0/R1 determined per AJCC 8th edition (proximal, distal, and circumferential margins where applicable). ASA, American Society of Anesthesiologists; BMI, body mass index; CEA, carcinoembryonic antigen; CA125, cancer antigen 125; LNR, lymph node ratio. Recorded comorbidities were extracted from available medical history fields using prespecified keyword rules and are presented descriptively. Elevated preoperative CEA and CA125 were defined as >5 ng/mL and >35 U/mL, respectively, according to the institutional laboratory reference ranges.

**Table 2:** Perioperative outcomes and adapted textbook outcome components according to intraoperative stress burden.

Characteristic	Overall *N* = 2352	Low Burden *N* = 1252	Intermediate Burden *N* = 718	High Burden *N* = 382	*p*-Value
Adapted textbook outcome					<0.001
Achieved	1732 (73.6%)	977 (78.0%)	506 (70.5%)	249 (65.2%)	
Failed	620 (26.4%)	275 (22.0%)	212 (29.5%)	133 (34.8%)	
Overall complications	413 (17.6%)	191 (15.3%)	130 (18.1%)	92 (24.1%)	<0.001
Severe complications (Clavien–Dindo ≥ III)	192 (8.2%)	87 (6.9%)	63 (8.8%)	42 (11.0%)	0.032
Infectious complications	284 (12.1%)	120 (9.6%)	92 (12.8%)	72 (18.8%)	<0.001
Postoperative length of stay, days	9 (7, 12)	8 (6, 11)	9 (7, 12)	10 (8, 14)	<0.001
Resection margin					0.027
R0	2336 (99.3%)	1248 (99.7%)	712 (99.2%)	376 (98.4%)	
R1/R2	16 (0.7%)	4 (0.3%)	6 (0.8%)	6 (1.6%)	
Total lymph nodes retrieved	37 (27, 49)	37 (27, 49)	38 (27, 49)	36 (26, 48)	0.334

Note: Values are *n* (%) or median (Q1, Q3). *p* values: Pearson χ^2^, Kruskal–Wallis, or Fisher’s exact (when expected count < 5). Median 37 retrieved nodes (IQR 27–49) is consistent with JGCA D2 or D2+ lymphadenectomy.

**Table 3:** Multivariable logistic regression analysis of factors associated with failure to achieve adapted textbook outcome.

Variable	Adjusted OR (95% CI)	*p* Value
Intraoperative stress burden		
Low burden	—	*p* for trend < 0.001
Intermediate burden	1.50 (1.19–1.87)	<0.001
High burden	1.68 (1.28–2.21)	<0.001
Sex		
Male	—	
Female	0.79 (0.63–0.98)	0.037
Age, years	1.03 (1.02–1.04)	<0.001
BMI, kg/m^2^	1.05 (1.02–1.08)	0.002
ASA physical status		
I–II	—	
III–IV	1.14 (0.90–1.44)	0.271
Preoperative albumin, g/L	0.99 (0.97–1.01)	0.363
Preoperative hemoglobin, g/L	1.00 (1.00–1.01)	0.614
Borrmann type		
Type I	—	
Type II	0.74 (0.46–1.22)	0.231
Type III	1.01 (0.65–1.63)	0.950
Type IV	1.28 (0.75–2.23)	0.365
Tumor location		
Upper	—	
Middle	0.68 (0.50–0.91)	0.010
Lower	0.69 (0.54–0.87)	0.002
Whole stomach	0.45 (0.27–0.73)	0.002
Differentiation grade		
Mod/Well	—	
Poor/Undiff	0.95 (0.77–1.17)	0.622
Pathologic T stage		
pT1–2	—	
pT3–4	0.94 (0.74–1.21)	0.647
Pathologic N stage		
pN0–1	—	
pN2–3	0.80 (0.64–0.99)	0.038
Surgical approach		
Open	—	
Minimally invasive	0.61 (0.49–0.77)	<0.001
Type of gastrectomy		
Distal	—	
Proximal	1.26 (0.71–2.19)	0.415
Total	0.91 (0.75–1.11)	0.345

Note: All variables in the table were mutually adjusted. Reference categories listed first; ‘—’ indicates reference. Minimally invasive includes laparoscopic and robotic; reference: open. aOR, adjusted odds ratio; CI, confidence interval; TO, adapted textbook outcome.

**Table 4:** Multivariable Cox proportional-hazards models for overall survival.

Characteristic	Model 1 aHR (95% CI)	*p*	Model 2 aHR (95% CI)	*p*	Model 3 aHR (95% CI)	*p*
Intraoperative stress burden						
Low burden	Reference		Reference		Reference	
Intermediate burden	1.07 (0.93–1.24)	0.326	1.05 (0.91–1.22)	0.490	1.06 (0.92–1.23)	0.397
High burden	1.36 (1.15–1.62)	<0.001	1.34 (1.13–1.59)	<0.001	1.32 (1.12–1.57)	0.001
*p* for trend		<0.001		0.002		0.003
Sex						
Male	Reference		Reference		Reference	
Female	1.11 (0.97–1.27)	0.119	1.12 (0.98–1.28)	0.096	1.11 (0.97–1.27)	0.129
Age, years	1.01 (1.01–1.02)	<0.001	1.01 (1.01–1.02)	<0.001	1.01 (1.00–1.02)	<0.001
BMI, kg/m^2^	1.00 (0.99–1.02)	0.637	1.00 (0.98–1.02)	0.865	1.00 (0.98–1.02)	0.748
ASA physical status						
I–II	Reference		Reference		Reference	
III–IV	0.97 (0.83–1.13)	0.691	0.97 (0.83–1.13)	0.658	0.99 (0.85–1.16)	0.910
Preoperative albumin, g/L	0.99 (0.98–1.01)	0.393	0.99 (0.98–1.01)	0.451	0.99 (0.98–1.01)	0.426
Preoperative hemoglobin, g/L	1.00 (1.00–1.00)	0.505	1.00 (1.00–1.00)	0.449	1.00 (1.00–1.00)	0.282
Borrmann type						
Type I	Reference		Reference		Reference	
Type II	0.62 (0.46–0.84)	0.002	0.62 (0.46–0.85)	0.002	0.61 (0.45–0.82)	0.001
Type III	0.75 (0.56–0.99)	0.042	0.74 (0.56–0.98)	0.036	0.71 (0.53–0.94)	0.018
Type IV	1.19 (0.86–1.65)	0.296	1.17 (0.84–1.62)	0.343	1.06 (0.77–1.48)	0.709
Tumor location						
Upper	Reference		Reference		Reference	
Middle	0.81 (0.66–0.98)	0.032	0.83 (0.68–1.01)	0.059	0.82 (0.68–1.00)	0.053
Lower	0.95 (0.81–1.11)	0.508	0.97 (0.83–1.14)	0.722	0.96 (0.82–1.13)	0.650
Whole stomach	1.22 (0.93–1.60)	0.144	1.27 (0.97–1.67)	0.083	1.29 (0.98–1.69)	0.067
Differentiation grade						
Moderately/Well differentiated	Reference		Reference		Reference	
Poorly/Undifferentiated	1.13 (0.99–1.28)	0.077	1.12 (0.98–1.28)	0.087	1.12 (0.98–1.27)	0.104
Pathologic T stage						
pT1–2	Reference		Reference		Reference	
pT3–4	1.73 (1.46–2.04)	<0.001	1.74 (1.47–2.06)	<0.001	1.76 (1.48–2.10)	<0.001
Pathologic N stage						
pN0–1	Reference		Reference		Reference	
pN2–3	1.85 (1.61–2.11)	<0.001	1.87 (1.63–2.13)	<0.001	1.56 (1.35–1.81)	<0.001
Surgical approach						
Open	Reference		Reference		Reference	
Minimally invasive	1.17 (1.01–1.35)	0.031	1.19 (1.03–1.38)	0.016	1.19 (1.03–1.38)	0.017
Type of gastrectomy						
Distal	Reference		Reference		Reference	
Proximal	0.98 (0.65–1.46)	0.908	0.98 (0.65–1.46)	0.915	0.73 (0.46–1.14)	0.169
Total	0.82 (0.72–0.94)	0.003	0.83 (0.73–0.94)	0.003	0.83 (0.73–0.94)	0.004
Adapted textbook outcome						
Achieved TO			Reference		Reference	
Failed TO			1.27 (1.11–1.46)	<0.001	1.24 (1.08–1.43)	0.002
Adjuvant chemotherapy						
No					Reference	
Yes					0.82 (0.72–0.94)	0.004
Lymph node ratio, per 0.1 increase					1.09 (1.07–1.12)	<0.001

Note: Model 1 adjusted for sex, age, BMI, ASA, preoperative albumin and hemoglobin, Borrmann type, tumor location, differentiation grade, pT, pN, surgical approach, and gastrectomy type. Model 2 adds adapted textbook outcome (TO). Model 3 adds adjuvant chemotherapy (Yes/No) and lymph node ratio (LNR, per 0.1 absolute increase). *N* = 2352; deaths = 1061. *p* for trend: stress burden coded as ordinal 0/1/2. Proportional-hazards assumption assessed by Schoenfeld residuals. References: low burden, male, ASA I–II, Borrmann I, upper tumor, mod/well differentiated, pT1–2, pN0–1, open surgery, distal gastrectomy, achieved TO, and no adjuvant chemotherapy. Minimally invasive includes laparoscopic and robotic cases. aHR, adjusted hazard ratio; CI, confidence interval; TO, adapted textbook outcome; LNR, lymph node ratio; BMI, body mass index; ASA, American Society of Anesthesiologists.

## Data Availability

The datasets used and/or analyzed during the current study, together with the analytic code, are available from the corresponding author on reasonable request.

## Supplementary Materials

The following supporting information can be downloaded at https://www.mdpi.com/article/10.3390/cancers18121975/s1, Figure S1: Kaplan–Meier curves for overall survival according to adapted textbook outcome status; Figure S2: Forest plot of the multivariable Cox model for overall survival in the full cohort; Figure S3: Forest plot of the sensitivity Cox analysis for overall survival in the no-transfusion cohort; Figure S4: Distribution of overlap weights across intraoperative stress burden groups. The plot shows the distribution of raw ATO overlap weights used in the weighted sensitivity analyses; Figure S5: Love plot of covariate balance before and after overlap weighting. The plot shows maximum absolute standardized mean differences across pairwise comparisons before and after ATO overlap weighting using baseline covariates only; the dashed vertical line indicates the 0.10 threshold for acceptable balance; Figure S6: Subgroup analyses of overall survival according to high versus low intraoperative stress burden; Table S1: Definitions of adapted textbook outcome and the intraoperative stress burden score; Table S2: Baseline characteristics of the study population after overlap weighting (ATO estimand); Table S3: Sensitivity analyses using overlap weighting (ATO); Table S4: Distribution of overlap weights and effective sample size under overlap weighting; Table S5: Sensitivity analyses additionally adjusted for operative time; Table S6: Sensitivity analyses additionally adjusted for surgical period; Table S7: Outcomes according to the original intraoperative stress burden score; Table S8: Proportional hazards assumption assessed using Schoenfeld residuals; Table S9: Time-interval-specific hazard ratios for overall survival; Table S10: Five-year time-dependent C-statistics for overall survival models; Table S11: Exploratory subgroup analyses of high versus low intraoperative stress burden for overall survival; Table S12: Incremental discrimination of intraoperative stress burden versus operative time; Table S13: Sensitivity analysis of the intraoperative stress burden–overall survival association using alternative thresholds; Table S14: Composite intraoperative stress burden score versus individual intraoperative components.
