# Supplementary material for: Intraoperative Stress Burden, Adapted Textbook Outcome, and Overall Survival After Curative-Intent Gastrectomy for Gastric Adenocarcinoma: A Single-Center Retrospective Cohort Study
Source: Cancers (Basel). 2026 Jun 18;18(12):1975. doi: 10.3390/cancers18121975 (PMC13296978; doi:10.3390/cancers18121975)
Supplement: Supplementary file 1 [file cancers-18-01975-s001.zip › cancers-4370387-supplementary.pdf]

Supplementary Materials

Intraoperative Stress Burden, Adapted Textbook Outcome, and Overall Survival After Curative-Intent Gastrectomy for Gastric Adenocarcinoma: A Single-Center Retrospective Cohort Study

Supplementary Table S1. Definitions of adapted textbook outcome and the intraoperative stress burden score.

Item	Achieved criterion or definition	Failed definition or scoring rule	Explanation
**A. Definitions and criteria for adapted textbook outcome (TO)**			
R0 resection	R0	R1/R2	Oncologically complete resection
Adequate lymph node retrieval	At least 15 lymph nodes retrieved	Fewer than 15 lymph nodes retrieved	Consistent with commonly used standards in curative gastric cancer surgery
No severe postoperative complication	No Clavien–Dindo grade III or higher complication	Any Clavien–Dindo grade III or higher complication	Major morbidity was defined as Clavien–Dindo grade III or higher, reflecting complications requiring surgical, endoscopic, radiologic, or intensive care intervention.
No reoperation	No unplanned reoperation during the index hospitalization or within 30 days after surgery	Any unplanned reoperation during the index hospitalization or within 30 days after surgery	Defined as no reoperation rather than no re-intervention
No death within 30 days	Alive within 30 days after surgery	Death within 30 days after surgery	Short-term safety indicator
No prolonged postoperative stay	Postoperative length of stay ≤12 days	Postoperative length of stay >12 days	Cohort-specific 75th percentile threshold, used to reflect prolonged postoperative stay under the local recovery pathway; not directly comparable with TO definitions using a 21-day threshold.
Final textbook outcome	All components achieved	Failure of any component	Achieved TO required simultaneous achievement of all six adapted TO components; failed TO was defined as failure of any component.
**B. Definitions and scoring rules for the intraoperative stress burden score**			

High blood loss burden	Intraoperative blood loss > 75th percentile of the cohort (P75 = 200 mL)	Yes = 1; No = 0	Used to identify upper-tail blood loss burden
High fluid burden	Intraoperative fluid administration > 75th percentile of the cohort (P75 = 68 mL/kg)	Yes = 1; No = 0	Used to identify higher fluid load exposure
Transfusion exposure	Any intraoperative blood product transfusion	Yes = 1; No = 0	Restricted to intraoperative transfusion; postoperative or non-intraoperative perioperative transfusion was not included in the score to preserve temporality.
Total score	Sum of the three components above	Range 0–3	Each component was coded as a binary item
Low burden	Total score = 0	Reference	Blood loss ≤ 200 mL, fluid administration ≤ 68 mL/kg, and no transfusion exposure
Intermediate burden	Total score = 1	—	Only one of the three adverse components was present
High burden	Total score = 2–3	—	Two or all three adverse components were present

Note: This study used an adapted TO definition based on prior TO frameworks, data availability, and the local postoperative recovery pathway. Thirty-day readmission and unplanned intensive care unit admission were not included because these variables were not systematically available in the database. The postoperative length-of-stay threshold was defined using the cohort-specific 75th percentile. The intraoperative stress burden score was designed to capture cumulative intraoperative burden rather than estimate the independent effect of each component.

Supplementary Table S2. Baseline characteristics of the study population after overlap weighting (ATO estimand).

Characteristic	Overall (unweighted N = 2,352)	Low burden (unweighted N = 1,252)	Intermediate burden (unweighted N = 718)	High burden (unweighted N = 382)	Max ASMD after ATO
Age, years	60.5 ± 11.9	60.5 ± 11.7	60.5 ± 12.3	60.5 ± 11.6	0.007
Body mass index, kg/m^2	21.3 ± 3.2	21.4 ± 3.1	21.3 ± 3.3	21.3 ± 3.2	0.030
Preoperative albumin, g/L	37.2 ± 5.2	37.3 ± 4.9	37.3 ± 5.1	37.2 ± 5.5	0.027
Preoperative hemoglobin, g/L	114.1 ± 25.4	115.2 ± 23.5	113.5 ± 26.9	113.7 ± 25.6	0.068
Sex					0.009
Male	1,573 (65.7%)	866 (65.8%)	461 (65.1%)	246 (66.0%)	
Female	779 (34.3%)	386 (34.2%)	257 (34.9%)	136 (34.0%)	
ASA class					0.006
I–II	1,877 (78.8%)	1,009 (78.5%)	570 (79.1%)	298 (78.7%)	
III–IV	475 (21.2%)	243 (21.5%)	148 (20.9%)	84 (21.3%)	
Borrmann type					0.006
Type I	101 (4.4%)	53 (4.6%)	32 (4.3%)	16 (4.4%)	
Type II	614 (21.3%)	392 (21.0%)	156 (21.3%)	66 (21.5%)	
Type III	1,421 (63.2%)	730 (63.4%)	442 (62.9%)	249 (63.4%)	
Type IV	216 (11.0%)	77 (10.9%)	88 (11.4%)	51 (10.8%)	
Tumor location					0.009
Upper	579 (28.0%)	298 (27.8%)	151 (28.2%)	130 (27.9%)	
Middle	477 (19.8%)	253 (19.7%)	156 (19.5%)	68 (20.4%)	
Lower	1,142 (47.0%)	599 (47.4%)	377 (46.9%)	166 (46.6%)	
Whole stomach	154 (5.2%)	102 (5.2%)	34 (5.4%)	18 (5.0%)	
Differentiation grade					0.013
Moderately/Well	1,097 (47.1%)	614 (47.2%)	293 (46.3%)	190 (47.6%)	
Poorly/Undifferentiated	1,255 (52.9%)	638 (52.8%)	425 (53.7%)	192 (52.4%)	
T stage					0.005
pT1–2	760 (22.5%)	505 (22.7%)	194 (22.2%)	61 (22.7%)	

pT3–4	1,592 (77.5%)	747 (77.3%)	524 (77.8%)	321 (77.3%)	
N stage					0.006
pN0–1	1,365 (52.9%)	773 (52.6%)	408 (53.2%)	184 (53.0%)	
pN2–3	987 (47.1%)	479 (47.4%)	310 (46.8%)	198 (47.0%)	
Surgical approach					0.009
Open	1,644 (74.7%)	794 (75.3%)	560 (74.4%)	290 (74.5%)	
Minimally invasive (laparoscopic or robotic)	708 (25.3%)	458 (24.7%)	158 (25.6%)	92 (25.5%)	
Gastrectomy type					0.015
Distal	1,274 (55.1%)	711 (55.5%)	346 (54.2%)	217 (55.6%)	
Proximal	60 (2.0%)	37 (1.9%)	16 (2.0%)	7 (2.1%)	
Total	1,018 (42.9%)	504 (42.6%)	356 (43.8%)	158 (42.3%)	

Note: Continuous variables are presented as weighted mean ± SD; categorical variables as unweighted n (weighted %).
Max ASMD after ATO indicates the maximum absolute standardized mean difference across all pairwise treatment comparisons after overlap weighting.
An absolute standardized mean difference <0.10 was considered indicative of adequate balance.

Supplementary Table S3. Sensitivity analyses using overlap weighting (ATO).

**Panel**	**Outcome**	**Comparison**	**OR/HR**	**95% CI**	**P value**
Panel A	TO failure	Intermediate burden vs Low burden	OR 1.55	1.21–1.98	<0.001
Panel A	TO failure	High burden vs Low burden	OR 1.70	1.27–2.26	<0.001
Panel B	Overall survival	Intermediate burden vs Low burden	HR 1.13	0.96–1.33	0.143
Panel B	Overall survival	High burden vs Low burden	HR 1.44	1.21–1.73	<0.001

Note: Panel A shows the results of the ATO-weighted logistic regression model for TO failure (N = 2,352). Panel B shows the results of the ATO-weighted Cox proportional hazards model for overall survival (N = 2,352; death events = 1,061).
Both models were additionally adjusted for the same baseline covariates used in the overlap weighting model: sex, age, body mass index, ASA physical status, preoperative albumin, preoperative hemoglobin, Borrmann type, tumor location, differentiation grade, pathologic T stage, pathologic N stage, surgical approach, and type of gastrectomy.
Low burden was used as the reference group.

Supplementary Table S4. Distribution of overlap weights and effective sample size under overlap weighting.

Group	Unweighted N	Sum of weights	Mean weight	SD	Median	IQR	Min	Max	ESS	ESS / N, %
Overall	2,352	511.343	0.217	0.156	0.187	0.099–0.292	0.005	0.896	1550.2	65.9
Low burden	1,252	169.669	0.136	0.104	0.109	0.058–0.187	0.005	0.643	790.6	63.1
Intermediate burden	718	170.688	0.238	0.081	0.229	0.176–0.287	0.077	0.484	644.1	89.7
High burden	382	170.986	0.448	0.166	0.440	0.320–0.573	0.090	0.896	335.9	87.9

Note: Values are presented for the overall cohort and for each intraoperative stress burden group separately.
Effective sample size (ESS) was calculated as (sum of weights)^2 divided by the sum of squared weights.
Overlap weighting targeted the average treatment effect in the overlap population (ATO).
ESS / N (%) represents the proportion of effective sample size relative to the unweighted sample size.

Supplementary Table S5. Sensitivity analyses additionally adjusted for operative time.

Model	Variable	Measure	Estimate	95% CI	P value
Panel A. TO failure model + operative time	Intermediate burden vs Low burden	OR	1.26	1.00–1.60	0.048
Panel A. TO failure model + operative time	High burden vs Low burden	OR	1.22	0.90–1.64	0.192
Panel A. TO failure model + operative time	Operative time, per 30-min increase	OR	1.12	1.08–1.16	<0.001
Panel B. OS Model 1a + operative time	Intermediate burden vs Low burden	HR	1.07	0.92–1.23	0.403
Panel B. OS Model 1a + operative time	High burden vs Low burden	HR	1.34	1.12–1.60	0.002
Panel B. OS Model 1a + operative time	Operative time, per 30-min increase	HR	1.01	0.98–1.03	0.557
Panel C. OS Model 3a + operative time	Intermediate burden vs Low burden	HR	1.06	0.91–1.23	0.469
Panel C. OS Model 3a + operative time	High burden vs Low burden	HR	1.30	1.09–1.56	0.004
Panel C. OS Model 3a + operative time	Operative time, per 30-min increase	HR	1.01	0.98–1.03	0.595

Note: Panel A used multivariable logistic regression for adapted TO failure. N = 2,352; TO failure events = 620.
Panels B and C used Cox proportional hazards models for overall survival. N = 2,352; death events = 1,061.
All models additionally adjusted for operative time, modeled per 30-minute increase.
Base covariates included sex, age, body mass index, ASA physical status, preoperative albumin, preoperative hemoglobin, Borrmann type, tumor location, differentiation grade, pathologic T stage, pathologic N stage, surgical approach, and type of gastrectomy.
Model 3a additionally included adapted TO status, adjuvant chemotherapy receipt, and lymph node ratio when these variables were available.
Abbreviations: CI, confidence interval; HR, hazard ratio; OR, odds ratio; OS, overall survival; TO, adapted textbook outcome.

Supplementary Table S6. Sensitivity analyses additionally adjusted for surgical period.

Model	Variable	Measure	Estimate	95% CI	P value
Panel A. TO failure model + surgical period	Intermediate burden vs Low burden	OR	1.44	1.15–1.81	0.002
Panel A. TO failure model + surgical period	High burden vs Low burden	OR	1.62	1.23–2.13	<0.001
Panel A. TO failure model + surgical period	Minimally invasive vs Open	OR	0.70	0.54–0.90	0.006
Panel A. TO failure model + surgical period	Surgical period 2014–2017 vs 2010–2013	OR	0.82	0.63–1.05	0.109
Panel A. TO failure model + surgical period	Surgical period 2018–2020 vs 2010–2013	OR	0.69	0.52–0.93	0.013
Panel B. OS Model 1b + surgical period	Intermediate burden vs Low burden	HR	1.09	0.94–1.26	0.245
Panel B. OS Model 1b + surgical period	High burden vs Low burden	HR	1.41	1.19–1.67	<0.001
Panel B. OS Model 1b + surgical period	Minimally invasive vs Open	HR	1.06	0.91–1.24	0.459
Panel B. OS Model 1b + surgical period	Surgical period 2014–2017 vs 2010–2013	HR	0.94	0.80–1.11	0.446
Panel B. OS Model 1b + surgical period	Surgical period 2018–2020 vs 2010–2013	HR	1.26	1.04–1.53	0.019
Panel C. OS Model 3b + surgical period	Intermediate burden vs Low burden	HR	1.09	0.94–1.26	0.261
Panel C. OS Model 3b + surgical period	High burden vs Low burden	HR	1.38	1.16–1.64	<0.001
Panel C. OS Model 3b + surgical period	Minimally invasive vs Open	HR	1.05	0.90–1.24	0.516
Panel C. OS Model 3b + surgical period	Surgical period 2014–2017 vs 2010–2013	HR	0.95	0.80–1.13	0.564
Panel C. OS Model 3b + surgical period	Surgical period 2018–2020 vs 2010–2013	HR	1.35	1.11–1.64	0.003

Note: Panel A used multivariable logistic regression for adapted TO failure. N = 2,352; TO failure events = 620.
Panels B and C used Cox proportional hazards models for overall survival. N = 2,352; death events = 1,061.
All models additionally adjusted for surgical period, categorized as 2010–2013, 2014–2017, and 2018–2020.
Base covariates included sex, age, body mass index, ASA physical status, preoperative albumin, preoperative hemoglobin, Borrmann type, tumor location, differentiation grade, pathologic T stage, pathologic N stage, surgical approach, and type of gastrectomy.
Model 3b additionally included adapted TO status, adjuvant chemotherapy receipt, and lymph node ratio.
Abbreviations: CI, confidence interval; HR, hazard ratio; OR, odds ratio; OS, overall survival; TO, adapted textbook outcome.

Supplementary Table S7. Outcomes according to the original intraoperative stress burden score.

Original stress score	N	Deaths	Adapted TO achieved	5-year OS	Adjusted HR for OS	P value
0	1,252	515	977/1252 (78.0%)	72.0% (69.5–74.7%)	Reference	—
1	718	330	506/718 (70.5%)	64.2% (60.6–67.9%)	1.08 (0.93–1.24)	0.323
2	279	155	189/279 (67.7%)	52.9% (47.1–59.4%)	1.31 (1.09–1.59)	0.005
3	103	61	60/103 (58.3%)	40.1% (30.9–52.0%)	1.51 (1.15–1.99)	0.003

Note: Adapted TO achieved indicates simultaneous achievement of all adapted textbook outcome components.
Five-year overall survival was estimated using the Kaplan–Meier method at 60 months.
Adjusted HRs were estimated using Cox proportional hazards models with stress score 0 as the reference.
The Cox model was adjusted for sex, age, body mass index, ASA physical status, preoperative albumin, preoperative hemoglobin, Borrmann type, tumor location, differentiation grade, pathologic T stage, pathologic N stage, surgical approach, and type of gastrectomy.
Abbreviations: CI, confidence interval; HR, hazard ratio; OS, overall survival; TO, adapted textbook outcome.

Supplementary Table S8. Proportional hazards assumption assessed using Schoenfeld residuals.

Variable	Chi_square	df	P value
Intraoperative stress burden	32.24	2	<0.001
sex	0.10	1	0.756
age	2.01	1	0.156
bmi	2.10	1	0.147
asa	0.01	1	0.926
alb_preop	0.40	1	0.526
hb_preop	2.90	1	0.088
borrmann	15.09	3	0.002
tumor_location	102.05	3	<0.001
diff_grade	17.21	1	<0.001
t_stage_group	40.75	1	<0.001
n_stage_group	13.99	1	<0.001
surgical_approach	110.45	1	<0.001
gastrectomy_type	0.51	2	0.775
Global test	245.84	20	<0.001

Note: The proportional hazards assumption was assessed using Schoenfeld residuals.
The main Cox model included intraoperative stress burden and the prespecified baseline covariates.
A P value <0.05 suggests evidence against the proportional hazards assumption.
Abbreviations: PH, proportional hazards.

Supplementary Table S9. Time-interval-specific hazard ratios for overall survival.

Time_interval	N at_risk	Deaths	Comparison	HR	95% CI	P value
0–12 months	2,352	128	Intermediate burden vs Low burden	1.55	1.00–2.38	0.049
0–12 months	2,352	128	High burden vs Low burden	2.03	1.25–3.27	0.004
12–60 months	2,182	613	Intermediate burden vs Low burden	1.20	0.99–1.46	0.057
12–60 months	2,182	613	High burden vs Low burden	1.54	1.24–1.92	<0.001
>60 months	1,250	320	Intermediate burden vs Low burden	0.77	0.59–1.00	0.053
>60 months	1,250	320	High burden vs Low burden	0.80	0.56–1.15	0.229

Note: Time intervals were defined as 0–12 months, 12–60 months, and >60 months after surgery.
Interval-specific HRs were estimated using Cox proportional hazards models within each time interval.
Models were adjusted for the same prespecified baseline covariates as the main Cox model.
The analysis was exploratory and intended to examine whether the association between intraoperative stress burden and overall survival varied over follow-up time.
Abbreviations: CI, confidence interval; HR, hazard ratio.

Supplementary Table S10. Five-year time-dependent C-statistics for overall survival models.

Model	5-year time-dependent C-statistic	Δ C-statistic vs baseline	Δ C-statistic vs burden model
Baseline covariates only	0.739	Reference	—
Baseline covariates + burden score	0.743	0.004	Reference
Baseline covariates + three components separately	0.744	0.005	0.001

Note: Five-year time-dependent C-statistics were estimated at 60 months using Cox model linear predictors. Complete-case N = 2,352; death events = 1,061.
Baseline covariates included sex, age, body mass index, ASA physical status, preoperative albumin, preoperative hemoglobin, Borrmann type, tumor location, differentiation grade, pathologic T stage, pathologic N stage, surgical approach, and type of gastrectomy.
The burden score model added categorical intraoperative stress burden group to the baseline covariate model.
The component model added high blood loss burden, high fluid burden, and intraoperative transfusion exposure separately to the baseline covariate model.
The purpose of this analysis was to evaluate incremental discrimination, not to establish a prediction model.

Supplementary Table S11. Exploratory subgroup analyses of high versus low intraoperative stress burden for overall survival.

Subgroup	Level	N (deaths)	High vs Low burden HR (95% CI)	P value	P for interaction
Pathologic T stage	pT1–2	566 (175)	0.95 (0.58–1.53)	0.819	0.335
Pathologic T stage	pT3–4	1068 (556)	1.50 (1.23–1.81)	<0.001	0.335
Pathologic N stage	pN0–1	957 (344)	1.13 (0.85–1.50)	0.415	0.075
Pathologic N stage	pN2–3	677 (387)	1.68 (1.34–2.11)	<0.001	0.075
Surgical approach	Open	1084 (502)	1.49 (1.22–1.83)	<0.001	0.119
Surgical approach	Minimally invasive	550 (229)	1.26 (0.86–1.86)	0.238	0.119
Adjuvant chemotherapy	No	844 (358)	1.67 (1.30–2.15)	<0.001	0.503
Adjuvant chemotherapy	Yes	790 (373)	1.25 (0.98–1.61)	0.074	0.503

Note: Subgroup analyses compared high intraoperative stress burden with low burden; intermediate burden patients were not included in this exploratory contrast. N and deaths refer to patients included in the high-versus-low burden contrast; intermediate-burden patients were not included in this exploratory subgroup contrast.
Cox models were adjusted for the same prespecified baseline covariates as the main Cox model, excluding the stratifying subgroup variable when applicable.
P for interaction was calculated by adding the interaction term between high burden and subgroup status to the Cox model.
These subgroup analyses were exploratory and were not adjusted for multiple comparisons.
Abbreviations: CI, confidence interval; HR, hazard ratio.

Supplementary Table S12. Incremental discrimination of intraoperative stress burden versus operative time.

Model	OS — 5-year time-dependent AUC (95% CI)	Adapted TO — AUC (95% CI)
Baseline covariates	0.739 (0.725–0.768)	0.658 (0.634–0.683)
Baseline + ISB	0.743 (0.729–0.771)	0.667 (0.643–0.691)
Baseline + operative time	0.740 (0.725–0.768)	0.685 (0.661–0.709)
Baseline + ISB + operative time	0.743 (0.729–0.771)	0.687 (0.663–0.711)

Note: Baseline covariates were the prespecified Model 1 covariates: sex, age, BMI, ASA physical status, preoperative albumin and hemoglobin, Borrmann type, tumor location, differentiation grade, pT stage, pN stage, surgical approach, and gastrectomy type. OS discrimination was assessed using the 5-year time-dependent cumulative/dynamic AUC with inverse-probability-of-censoring weighting. Adapted TO AUC was calculated from logistic models for TO failure. Operative time was modeled per 30-minute increment. Likelihood-ratio tests for OS: ISB | baseline, $\chi^2 = 12.33$ (2 df), P = 0.002; operative time | baseline, $\chi^2 = 2.78$ (1 df), P = 0.095; ISB | baseline + operative time, $\chi^2 = 9.90$ (2 df), P = 0.007; operative time | baseline + ISB, $\chi^2 = 0.34$ (1 df), P = 0.558. Likelihood-ratio tests for adapted TO failure: ISB | baseline, $\chi^2 = 18.78$ (2 df), P < 0.001; operative time | baseline, $\chi^2 = 51.83$ (1 df), P < 0.001; ISB | baseline + operative time, $\chi^2 = 4.14$ (2 df), P = 0.126; operative time | baseline + ISB, $\chi^2 = 37.19$ (1 df), P < 0.001. ISB, intraoperative stress burden; OS, overall survival; TO, adapted textbook outcome; AUC, area under the curve.

Supplementary Table S13. Sensitivity analysis of the intraoperative stress burden–overall survival association using alternative thresholds.

Threshold scheme	Blood loss	Fluid	n (low/intermediate/high)	Intermediate HR (95% CI)	High HR (95% CI)
Primary (75th percentile)	>200 mL	>68 mL/kg	1252/718/382	1.07 (0.93–1.24)	1.36 (1.15–1.62)
Median (50th percentile)	>100 mL	>56 mL/kg	664/898/790	1.01 (0.86–1.18)	1.25 (1.06–1.47)
90th percentile	>400 mL	>81 mL/kg	1658/512/182	1.36 (1.17–1.58)	1.52 (1.22–1.89)
Fixed clinical cutoffs	>500 mL	>75 mL/kg	1593/587/172	1.28 (1.11–1.49)	1.57 (1.26–1.95)

Note: All models were multivariable Cox models adjusted for the prespecified Model 1 covariates, with the low-burden group as the reference. The transfusion component, defined as any intraoperative transfusion, was unchanged across threshold schemes. High burden was defined as a score of 2–3. HR, hazard ratio; CI, confidence interval; ISB, intraoperative stress burden; OS, overall survival.

Supplementary Table S14. Composite intraoperative stress burden score versus individual intraoperative components.

Analysis	Endpoint	Model / comparison / component	Statistic	Estimate	df	P value
Model discrimination	Overall survival	Baseline covariates	AUC	0.739		
		Baseline + composite ISB score	AUC	0.743		
		Baseline + three ISB components	AUC	0.744		
	Adapted TO failure	Baseline covariates	AUC	0.658		
		Baseline + composite ISB score	AUC	0.667		
		Baseline + three ISB components	AUC	0.668		
Likelihood-ratio test	Overall survival	Composite ISB score | baseline	χ^2	12.33	2	0.002
		Three components | baseline	χ^2	20.70	3	<0.001
	Adapted TO failure	Composite ISB score | baseline	χ^2	18.78	2	<0.001
		Three components | baseline	χ^2	19.66	3	<0.001
Component association	Overall survival	High blood loss	Adjusted HR (95% CI)	1.07 (0.92–1.25)		0.386
		High fluid administration	Adjusted HR (95% CI)	1.01 (0.87–1.17)		0.941
		Any intraoperative transfusion	Adjusted HR (95% CI)	1.41 (1.19–1.68)		<0.001
	Adapted TO failure	High blood loss	Adjusted OR (95% CI)	1.23 (0.97–1.56)		0.088
		High fluid administration	Adjusted OR (95% CI)	1.39 (1.10–1.76)		0.005
		Any intraoperative transfusion	Adjusted OR (95% CI)	1.29 (0.98–1.70)		0.071

Baseline covariates were the prespecified Model 1 covariates: sex, age, BMI, ASA physical status, preoperative albumin and hemoglobin, Borrmann type, tumor location, differentiation grade, pT stage, pN stage, surgical approach, and gastrectomy type. The composite ISB score was modeled as low, intermediate, and high burden, with low burden as the reference. The component model included high blood loss, high fluid administration, and any intraoperative transfusion simultaneously, without additionally including the composite ISB score. Overall-survival discrimination was assessed using the 5-year IPCW cumulative/dynamic AUC. Adapted TO AUC was calculated from logistic models for TO failure. HR and OR estimates for the component model are mutually adjusted for the other two components and baseline covariates. ISB, intraoperative stress burden; OS, overall survival; TO, adapted textbook outcome; IPCW, inverse-probability-of-censoring weighting; AUC, area under the curve; HR, hazard ratio; OR, odds ratio; CI, confidence interval.

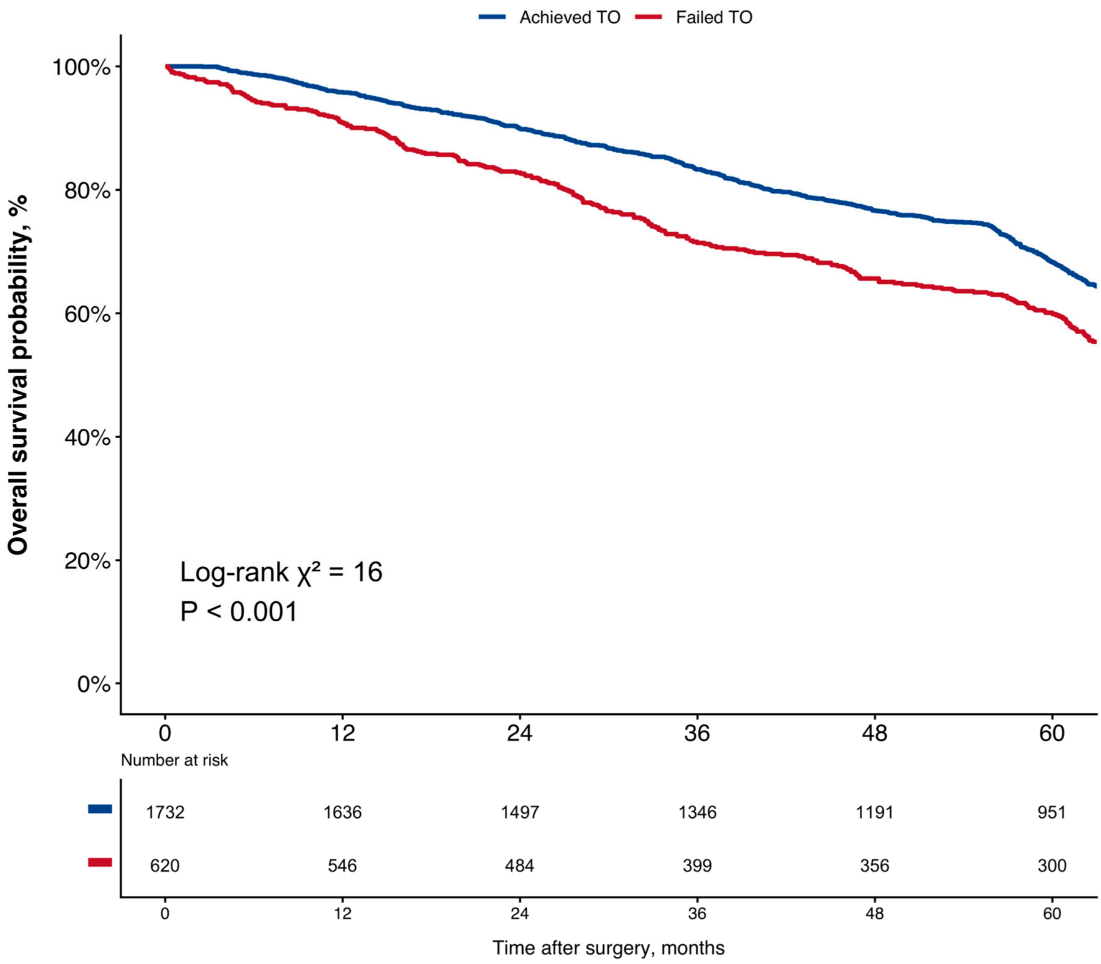

Supplementary Figure S1. Kaplan–Meier curves for overall survival according to adapted textbook outcome status.

Variable		N	aHR (95% CI)	P value
**Burden**	Low burden	1252	Reference	
	Intermediate burden	718	1.06 (0.92, 1.23)	0.397
	High burden	382	1.32 (1.12, 1.57)	0.001
**Sex**	Male	1573	Reference	
	Female	779	1.11 (0.97, 1.27)	0.129
**Age, years**		2352	1.01 (1.00, 1.02)	<0.001
**Body mass index, kg/m²**		2352	1.00 (0.98, 1.02)	0.748
**ASA**	I-II	1877	Reference	
	III-IV	475	0.99 (0.85, 1.16)	0.910
**Preoperative albumin, g/L**		2352	0.99 (0.98, 1.01)	0.426
**Preoperative hemoglobin, g/L**		2352	1.00 (1.00, 1.00)	0.282
**Borrmann**	Type I	101	Reference	
	Type II	614	0.61 (0.45, 0.82)	0.001
	Type III	1421	0.71 (0.53, 0.94)	0.018
	Type IV	216	1.06 (0.77, 1.48)	0.709
**Location**	Upper	579	Reference	
	Middle	477	0.82 (0.68, 1.00)	0.053
	Lower	1142	0.96 (0.82, 1.13)	0.650
	Whole stomach	154	1.29 (0.98, 1.69)	0.067
**Differentiation**	Moderately/well differentiated	1097	Reference	
	Poorly/undifferentiated	1255	1.12 (0.98, 1.27)	0.104
**pT_Stage**	pT1–2	760	Reference	
	pT3–4	1592	1.76 (1.48, 2.10)	<0.001
**pN_Stage**	pN0–1	1365	Reference	
	pN2–3	987	1.56 (1.35, 1.81)	<0.001
**Approach**	Open	1644	Reference	
	Minimally invasive	708	1.19 (1.03, 1.38)	0.017
**Gastrectomy**	Distal	1274	Reference	
	Proximal	60	0.73 (0.46, 1.14)	0.169
	Total	1018	0.83 (0.73, 0.94)	0.004
**TO**	Achieved TO	1732	Reference	
	Failed TO	620	1.24 (1.08, 1.43)	0.002
**Chemo**	No	1179	Reference	
	Yes	1173	0.82 (0.72, 0.94)	0.004
**Lymph node ratio, per 0.1 increase**		2352	1.09 (1.07, 1.12)	<0.001

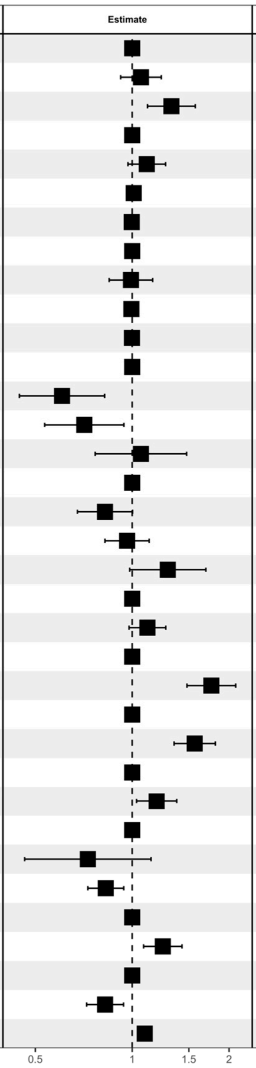

Supplementary Figure S2. Forest plot of the multivariable Cox model for overall survival in the full cohort.

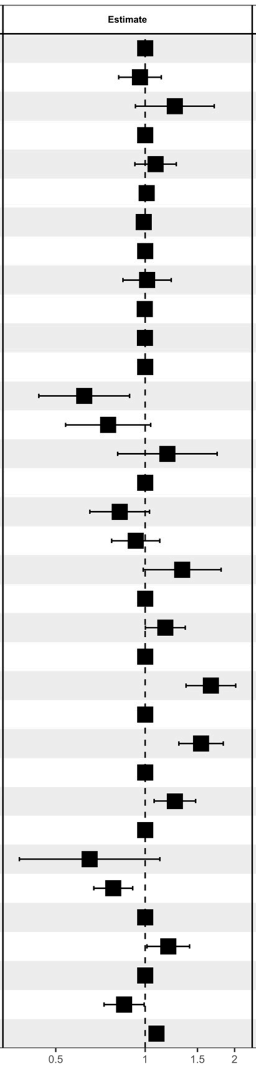

Variable		N	aHR (95% CI)	P value
**Burden**	Low burden	1252	Reference	
	Intermediate burden	531	0.96 (0.81, 1.13)	0.633
	High burden	94	1.26 (0.93, 1.71)	0.140
**Sex**	Male	1255	Reference	
	Female	622	1.08 (0.92, 1.27)	0.325
**Age, years**		1877	1.01 (1.00, 1.02)	0.001
**Body mass index, kg/m²**		1877	0.99 (0.97, 1.01)	0.338
**ASA**	I-II	1530	Reference	
	III-IV	347	1.01 (0.84, 1.22)	0.876
**Preoperative albumin, g/L**		1877	1.00 (0.98, 1.01)	0.699
**Preoperative hemoglobin, g/L**		1877	1.00 (0.99, 1.00)	0.373
**Borrmann**	Type I	78	Reference	
	Type II	528	0.62 (0.44, 0.89)	0.008
	Type III	1115	0.75 (0.54, 1.04)	0.088
	Type IV	156	1.19 (0.81, 1.75)	0.382
**Location**	Upper	448	Reference	
	Middle	379	0.82 (0.65, 1.03)	0.094
	Lower	922	0.93 (0.77, 1.12)	0.442
	Whole stomach	128	1.33 (0.99, 1.80)	0.063
**Differentiation**	Moderately/well differentiated	873	Reference	
	Poorly/undifferentiated	1004	1.17 (1.00, 1.36)	0.048
**pT_Stage**	pT1–2	684	Reference	
	pT3–4	1193	1.66 (1.37, 2.02)	<0.001
**pN_Stage**	pN0–1	1127	Reference	
	pN2–3	750	1.54 (1.30, 1.83)	<0.001
**Approach**	Open	1265	Reference	
	Minimally invasive	612	1.26 (1.07, 1.48)	0.005
**Gastrectomy**	Distal	1021	Reference	
	Proximal	48	0.65 (0.38, 1.12)	0.121
	Total	808	0.78 (0.67, 0.91)	0.001
**TO**	Achieved TO	1423	Reference	
	Failed TO	454	1.20 (1.01, 1.41)	0.034
**Chemo**	No	958	Reference	
	Yes	919	0.85 (0.73, 0.99)	0.039
**Lymph node ratio, per 0.1 increase**		1877	1.09 (1.07, 1.12)	<0.001

Supplementary Figure S3. Forest plot of the sensitivity Cox analysis for overall survival in the no-transfusion cohort.

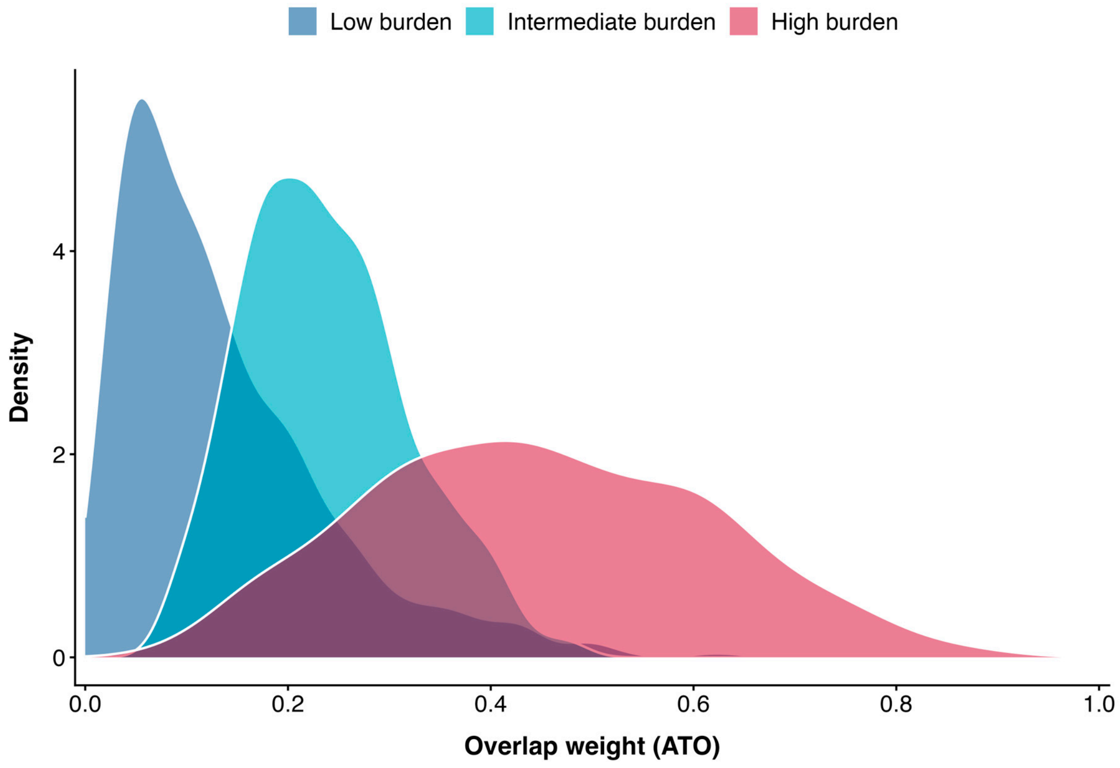

Supplementary Figure S4. Distribution of overlap weights across intraoperative stress burden groups. The plot shows the distribution of raw ATO overlap weights used in the weighted sensitivity analyses.

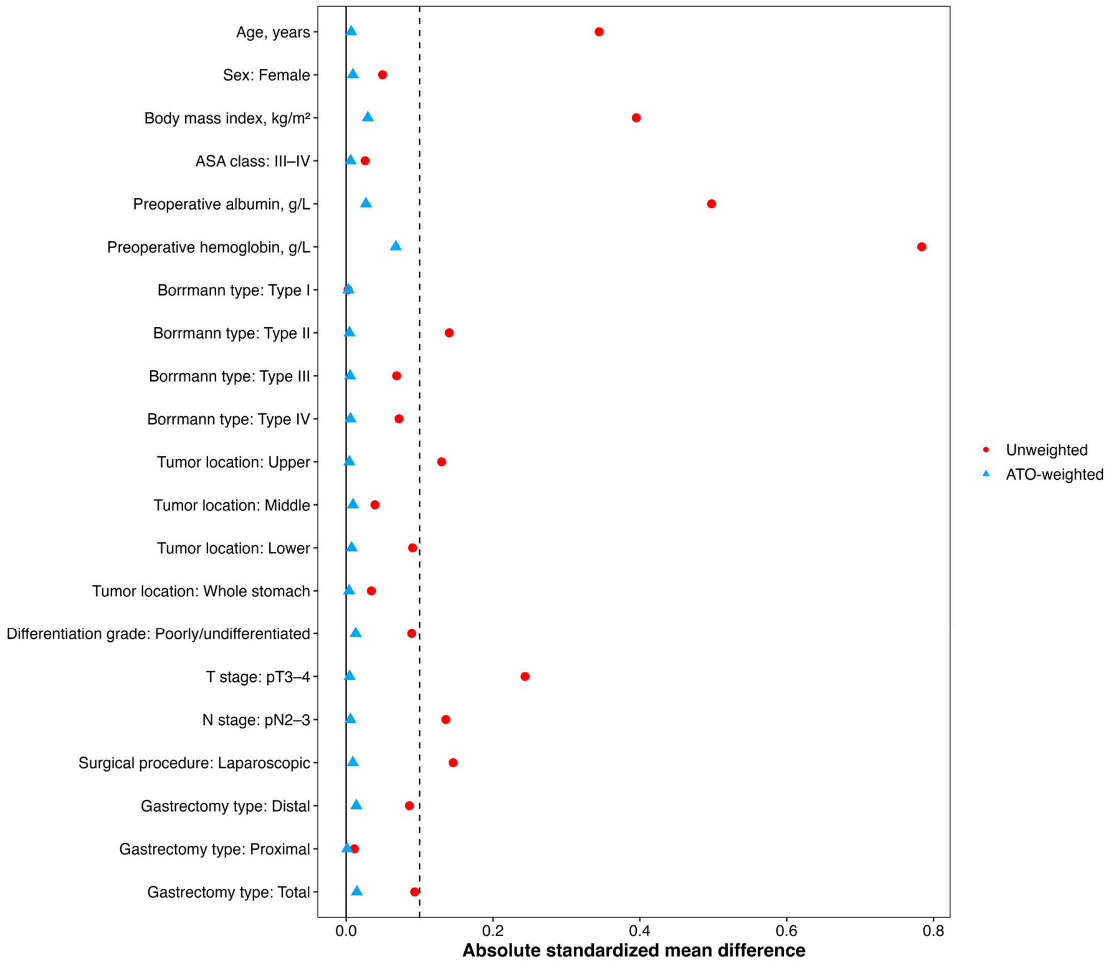

Supplementary Figure S5. Love plot of covariate balance before and after overlap weighting. The plot shows maximum absolute standardized mean differences across pairwise comparisons before and after ATO overlap weighting using baseline covariates only; the dashed vertical line indicates the 0.10 threshold for acceptable balance.

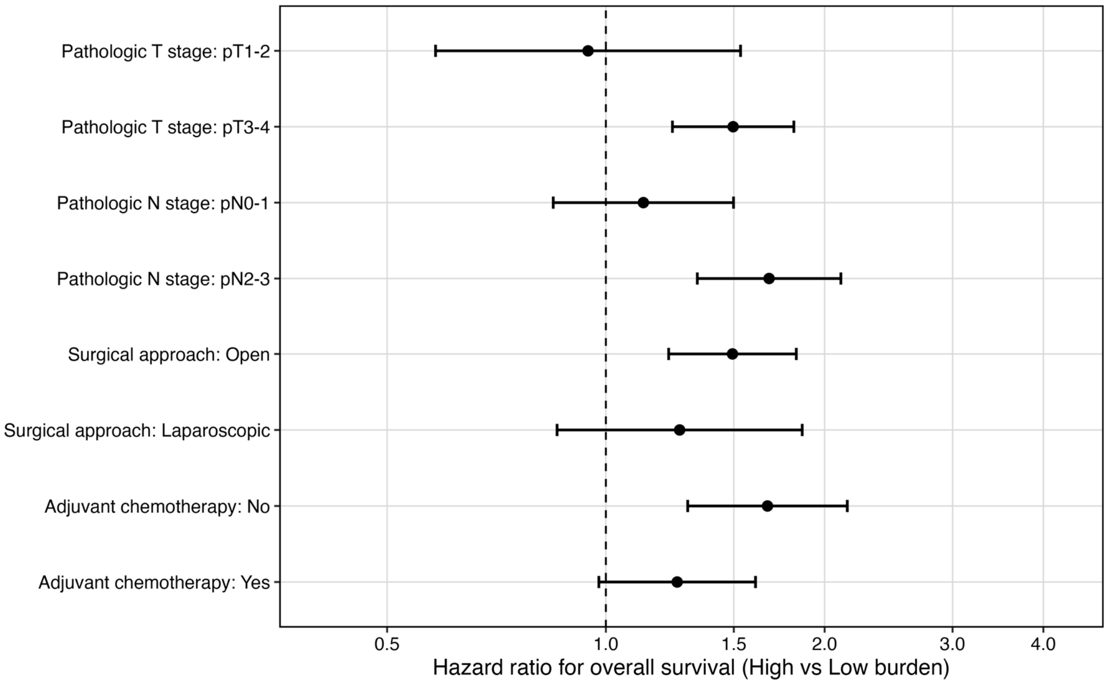

Supplementary Figure S6. Subgroup analyses of overall survival according to high versus low intraoperative stress burden.

Note: Forest plot showing adjusted hazard ratios for overall mortality (high vs low intraoperative stress burden) within prespecified clinical subgroups. Intermediate burden patients were not included in this exploratory contrast.

Each Cox model was adjusted for the same prespecified baseline covariates as the main Cox model, excluding the stratifying subgroup variable when applicable.

Solid circles indicate point estimates; horizontal bars indicate 95% confidence intervals; the vertical dashed line indicates a hazard ratio of 1.0. The x-axis is on a logarithmic scale.

All P values for interaction were >0.05 (Supplementary Table S11).
